# *Iota*-Carrageenan/Chitosan Nanoparticles via Coacervation: Achieving Stability for Tiny Particles

**DOI:** 10.3390/nano15030161

**Published:** 2025-01-22

**Authors:** Rosecler S. Klein, Débora A. de Almeida, Ariel C. de Oliveira, Elton G. Bonafé, Johny P. Monteiro, Roberta M. Sabino, Alessandro F. Martins

**Affiliations:** 1 Laboratory of Materials, Macromolecules, and Composites, Federal University of Technology-Paraná (UTFPR), Apucarana 86812-460, PR, Brazil; roseclerklein@alunos.utfpr.edu.br (R.S.K.); pg54822@uem.br (D.A.d.A.); cola2005@usherbrooke.ca (A.C.d.O.); eltonbonafe@utfpr.edu.br (E.G.B.); johnymonteiro@utfpr.edu.br (J.P.M.); 2 Department of Chemistry, State University of Maringá (UEM), Maringá 87020-900, PR, Brazil; 3 Biomass, Bioproduct and Bioprocess Analysis, Department of Chemical Engineering and Biotechnology Engineering, Université de Sherbrooke, 3000 Boul. de Université de Sherbrooke, Sherbrooke, QC J1K 0A5, Canada; 4 Department of Chemical and Biomedical Engineering, University of Wyoming (UW), Laramie, WY 82071, USA; 5 National Institute for Materials Advancement (NIMA), Pittsburg State University (PSU), Pittsburg, KS 66762, USA; 6 Department of Chemistry, Pittsburg State University (PSU), Pittsburg, KS 66762, USA

**Keywords:** polyelectrolytes, polysaccharides, nanomaterials, coacervates

## Abstract

This study investigated the influence of parameters such as pH condition, polyelectrolyte concentration, polymer ratio, and order of addition of the commercial polyelectrolytes chitosan and *iota*-carrageenan (ι-carrageenan) on the formation of polymeric nanoparticles in suspension (coacervates). A preliminary purification step of the polymers was essential for obtaining stable nanoparticles with small sizes as impurities, particularly metal ions that interfere with complexation, are removed by dialysis. Microparticles (13.5 μm in dry diameter) are obtained when aliquots of chitosan solution are poured into the ι-carrageenan solution. In general, an excess of chitosan results in the formation of agglomerated particles. The addition of an aliquot of ι-carrageenan solution (30 mL at 0.6 mg/mL and pH 4.0) to the chitosan solution (6.0 mL at 0.3 mg/mL and pH 4.0) leads to dispersed nanoparticles with a hydrodynamic radius of 278 ± 5 nm, a zeta potential of −31 ± 3 mV, and an average dry diameter of 45 ± 11 nm. The hydrodynamic radius increases as the pH rises. The partial deprotonation of ι-carrageenan chains enhances the interaction with water molecules, causing the particles to swell. These findings contribute to the fundamental understanding of polyelectrolyte complexation processes in aqueous suspension and provide insights for developing stable nanomaterials for potential practical applications.

## 1. Introduction

Polysaccharides with ionizable groups are usually classified as cationic and anionic polyelectrolytes [1]. Polyelectrolytes with acidic ionizable groups are polyanions and include the majority of polysaccharides, such as glycosaminoglycans (heparin, chondroitin sulfate, hyaluronic acid, and others), pectin, alginate, carrageenans, etc. These anionic polyelectrolytes are partially ionized in aqueous media at pH above 4.5, promoting groups with negative charge density [2]. Polysaccharides that exhibit positive charge density in aqueous media, such as chitosan, are called polycations, as they have basic protonated groups in dilute acid solutions [3].

Mixing polymeric solutions of polycations and polyanions can lead to the formation of nanoparticles in suspension (coacervates). This particle preparation method is commonly called complex coacervation or polyelectrolyte coacervation. Coacervates are obtained under specific conditions of pH, ionic strength, concentration, temperature, order of addition, mixing rate, and mass/mass ratio of polycation/polyanion, among others. Coacervates are mainly maintained by Coulombic interactions established between the oppositely charged ionizable groups of the polyelectrolytes. However, coacervate formation is also driven by hydrogen bonding and hydrophobic interactions [4].

The coacervation method has gained attention for promoting the preparation of small and stable physical nanoparticles. It is a simple, low-cost, and reproducible method that does not require energy, organic solvents, chemical cross-linkers, or surfactants [4,5]. Therefore, coacervates (suspended particles) are of significant interest as potential carriers for bioactive molecules such as drugs, proteins, and nucleic acids [4].

Coacervates are generally produced by combining biopolymers (polysaccharides), such as chitosan, with polyanions [6]. Chitosan stands out as the only commercially available cationic polysaccharide. Its high availability is due to its synthesis and precursor. Chitosan is mainly obtained by the partial deacetylation of chitin in an alkaline medium. Chitin is the second most abundant polysaccharide on the planet [7]. Chitosan has an amino group in each repeating unit of its D-glucosamine structure, resembling the chemical structure of cellular proteins, giving it a high capacity to establish supramolecular interactions with biomaterials. Due to these properties, chitosan-based drug delivery systems (polymeric nanoparticles) have received significant attention [4,7,8]. Additionally, the cationic nature of chitosan has been conveniently explored to form pH-responsive coacervates with anionic polysaccharides such as gums [9], pectin [10], *kappa*-carrageenan [11], and dextran sulfate [12,13,14,15].

Carrageenans are sulfated and water-soluble polysaccharides extracted from red algae (Rhodophyta). Their chemical structure consists of alternating β-D-galactose and α-D-galactose units linked by β-(1,4) and α-(1,3) bonds. Carrageenans are classified into three primary structural forms: kappa (κ), iota (ι), and lambda (λ). The primary difference between these forms lies in the structure and position of the disaccharide units (galactose and anhydrogalactose) [16]. ι-Carrageenan, the focus of this study, has two sulfate groups per repeating unit. These sulfate groups are ionized in aqueous solutions with a pH above 2.6, allowing for the complexation of ι-carrageenan with chitosan. ι-Carrageenan exhibits many properties, such as anticancer [17], antiviral [18], and anticoagulant activities [19], and bone promotion [20], wound healing, and antimicrobial treatment [21].

Although there are published articles on chitosan and κ-carrageenan coacervates [11], there is a lack of knowledge about chitosan and ι-carrageenan coacervates. Additionally, to date, there are no studies on the production of chitosan and ι-carrageenan coacervates that consider the influence of experimental parameters (pH, concentration, polycation/polyanion ratio, order of addition, i.e., addition of polycation to polyanion solution or vice versa, among others) that directly affect the properties of the resulting particles, especially for achieving stability and small particle sizes with average dry diameters below 100 nm. Therefore, it is necessary to deepen the understanding of the experimental protocols employed and systematically control the polyelectrolyte complexation to obtain nanomaterials.

Thus, the preparation of chitosan and ι-carrageenan coacervate nanoparticles with small sizes is reported in detail. Nano-coacervates were obtained under different pH conditions (3.6, 4.0, and 4.4) by adding ι-carrageenan solutions to chitosan solutions in a single step. The volume-to-volume ratio of the mixed polyelectrolyte solutions must be controlled to promote stable nano-coacervates in suspension. However, micro-coacervates are obtained when the chitosan solution is added to the ι-carrageenan solution, i.e., when the mixing order is reversed. These experimental factors are adjusted and controlled, resulting in stable nanoparticles with a low polydispersity index. The commercial polyelectrolytes chitosan and ι-carrageenan were characterized by molecular weight measurements using DLS, and the presence of metallic contaminants by Flame Atomic Absorption Spectroscopy (FAAS), infrared spectroscopy (FTIR), and differential scanning calorimetry (DSC). The coacervates were extensively characterized by DLS measurements such as hydrodynamic radius and zeta potential, SEM, FTIR, and DSC. This study demonstrates that precise control of experimental parameters is essential for producing stable and tiny polymeric nanomaterials in suspension.

## 2. Materials and Methods

### 2.1. Materials

Chitosan with 85% deacetylation was acquired from Golden-shell Biochemical (Shanghai, China). ι-Carrageenan was provided by CP Kelco (Limeira, São Paulo, Brazil). Other reagents, including glacial acetic acid, sodium acetate, methanol, ethanol, and acetone, were obtained from Dinâmica (São Paulo, Brazil).

### 2.2. Purification of Precursor Polymers

Solutions of chitosan and ι-carrageenan (1.0% *w*/*v*) were prepared in a dilute aqueous solution of acetic acid (1.0% *v*/*v*). These solutions were dialyzed in deionized water using cellulose membranes with a 12 kDa molecular weight cutoff for 6 days, with daily water changes. After dialysis, the solutions were frozen and lyophilized for 72 h at −60 °C using an SL 404 Solab freeze dryer (São Paulo, Brazil).

### 2.3. Characterization of Precursor Polymers

The polysaccharide samples (before and after purification) were extensively characterized. The samples were degraded in an oven at 600 °C for 6 h. Subsequently, 30 mL of 5.0% *v*/*v* HNO_3_ was added to each sample, and the resulting solution was filtered and analyzed using a Thermo Scientific ICE 3000 flame atomic absorption spectrometer (FAAS) (Thermo Scientific, Waltham, MA, USA). The concentrations of metallic ions, including magnesium (Mg^2+^), calcium (Ca^2+^), and sodium (Na^+^), were determined in the samples using appropriately diluted standard solutions. The results were expressed in milligrams of metallic ions per gram of each polysaccharide (mg/g).

The average molecular weight (${\bar{M}}_{w}$) of the polymers was determined through static light scattering (SLS) measurements using an Anton Paar Litesizer 500 (Graz, Austria), employing quartz cuvettes. Initially, aqueous solutions of the polymers were prepared in the concentration range of 0.125 to 2.0 mg/mL. Chitosan solutions were prepared in a 1.0% *v*/*v* acetic acid solution, while the ι-carrageenan solutions were obtained in deionized water. Before evaluating the ${\bar{M}}_{w}$, the refractive index of the polymer solutions was measured indirectly using substances with known refractive indices, such as acetone (1.3594), ethanol (1.3577), and methanol (1.3238). After recording the refractive index of the selected solvents, the refractive index of the polymer solutions was indirectly measured using the standard refractive index of the solvents. The change in polymer solution concentration provides the *dc* value, while the *dn* value is the refractive index difference that depends on the concentration and composition of the polymer solution.

These measurements were used to determine the refractive index increment (dn/dc) for polymer solutions within a concentration range of 0.125 to 2.0 mg/mL. The dn/dc value indicates the change in the solution’s refractive index per unit increase in polymer concentration. This parameter is used to calculate an optical constant, *K*, as defined in Equation (1), which is for determining molecular weight [22,23].

(1) $$K=4\;\pi^{2\;}{\left(\frac{dn}{dc}\right)}^{2}\frac{n_{0}^{2}}{N_{A}\lambda_{0}^{4}}$$

where *n*_0_ is the refractive index of the solvent, *N_A_* is the Avogadro constant, and *λ*_0_ is the wavelength of the incident beam (658 nm). The constant *K* relates to the polymer’s molar mass through the Debye–Zimm Equation (2) [24].

(2) $$\frac{KC}{R}=\frac{1}{{\bar{M}}_{w}}+2A_{2}C+...$$

where *C* is the concentration of polymer solutions (mg/mL), Δ*R* is the Rayleigh ratio of a reference solvent (toluene), ${\bar{M}}_{w}$ is the average molecular weight of the polymer in solution, and *A*_2_ is the virial coefficient.

To determine ${\bar{M}}_{w}$ and *A*_2_, measurements of the ratio *KC*/*R* were obtained as a function of the concentration (*C*) of the polymer solutions in the range of 0.125 to 2.0 mg/mL. Then, the results of ${\bar{M}}_{w}$ were obtained by performing measurements with the solvents used in the preparation of the polymer solutions (distilled water for ι-carrageenan and 1.0% *v*/*v* acetic acid for chitosan) and toluene, which was used to subtract the background scattering signal from the solutions. The values of ${\bar{M}}_{w}$ and *A*_2_ were obtained using Kalliope^®^ software 2.16.0 exe through Debye plot graphics. ${\bar{M}}_{w}$ is related to the linear coefficient of the *KC/R* curve as a function of concentration, *C*. In contrast, the slope is related to the coefficient *A*_2_.

The polymers were characterized by attenuated total reflectance Fourier-transform infrared spectroscopy (FTIR-ATR) using a Shimadzu Scientific Instruments 8300 spectrophotometer (Kyoto, Japan). Spectra were obtained in the 400 to 4000 cm^−1^ range with a resolution of 4 cm^−1^ and 64 scans. The materials were also characterized by differential scanning calorimetry (DSC) using a Shimadzu DSC60-Plus calorimeter (Kyoto, Japan) operating under the following conditions: heating rate of 10 °C/min, nitrogen gas flow of 50 mL/min, and temperature range of 25 to 350 °C. These same materials were analyzed using a Shimadzu TG50 thermogravimetric analyzer (Kyoto, Japan) under the following conditions: a heating rate of 10 °C/min, nitrogen gas flow at 50 mL/min, and a temperature range of 25 to 700 °C.

### 2.4. Preparation of Polymeric Nanoparticles

From this point, only the dialyzed (purified) polymers were used. Aqueous solutions of ι-carrageenan and chitosan were individually prepared at different concentrations in an acetic acid/sodium acetate buffer (0.1 M) at various pH conditions (3.6, 4.0, and 4.4) and filtered through 0.45 mm polytetrafluoroethylene filters (Fisher Scientific, Waltham, MA, USA). Then, the solutions were mixed, instantly pouring aliquots of the anionic polyelectrolyte solution (ι-carrageenan) into solutions of the cationic polyelectrolyte (chitosan) (Table 1) or pouring aliquots of chitosan solutions into ι-carrageenan solutions (Table 2). The volume-to-volume ratio of the mixtures was adjusted to polyanion/polyanion ratios (Table 1) or polycation/polyanion ratios (Table 2) of 2, 5, and 8. The mixtures were obtained and kept under magnetic stirring (750 rpm) for 1 h at room temperature. After this period, the mixtures (suspensions containing nanoparticulate material) were maintained at 4 °C for 12 h to precipitate larger particles from the obtained suspensions. Subsequently, the suspensions were centrifuged at 3000× *g* rpm for 10 min to ensure the removal of larger particles (precipitates) and stored at 4 °C for further use.

### 2.5. Characterization of the Nanoparticles

The hydrodynamic radius (nm) and zeta potential (mV) of the suspensions were determined using DLS measurements, employing the Anton Paar Litesizer 500 equipment (Graz, Austria) conducted at 25 °C and 37 °C. The hydrodynamic radius of the particles in suspension was obtained in a polystyrene cuvette. An omega (*Ω*)-shaped cuvette was used for zeta potential measurement.

The morphology and average diameter of the particles were assessed through scanning electron microscopy (SEM) images using an FEI Quanta 250 instrument (Prague, Czech Republic). Aliquots of 10 μL of particle suspensions were deposited onto polystyrene films, and the resulting system was placed in an oven at 25 °C for solvent evaporation. Subsequently, the samples were sputter-coated for 60 s with a thin layer of gold approximately 10 nm thick. SEM images were obtained by applying an electron beam acceleration voltage between 10 and 15 kV. From the SEM images, size distribution curves of 30 particles were obtained using ImageJ 1.8.0 software. Larger particles precipitating after centrifugation (Section 2.4) were characterized using FTIR-ATR and DSC, employing experimental conditions similar to those described in Section 2.3.

#### Hydrodynamic Radius Measurements as a Function of pH

Nanoparticles of ι-carrageenan/chitosan were obtained following the conditions described in Experiment 7 (Table 1), with a slight modification. The nanoparticles were produced from aqueous solutions of the polysaccharides at pH 3.6 and adjusted using acetic acid. Separate aqueous solutions were prepared with purified ι-carrageenan at 0.6 mg/mL (30 mL) and purified chitosan at 0.3 mg/mL (6 mL), both filtered through 0.45 µm polytetrafluoroethylene membranes (Fisher Scientific). The solutions were then combined by the rapid addition of aliquots of the anionic polyelectrolyte solution (purified ι-carrageenan) to the cationic polyelectrolyte solution (purified chitosan). The resulting mixtures were stirred at 750 rpm for 1 h at room temperature. After this period, the nanoparticle suspensions were stored at 4 °C for 12 h to allow for the precipitation of larger particles. Subsequently, the suspensions were centrifuged at 3000× *g* rpm for 10 min to ensure the removal of larger particles (precipitates) and then stored at 4 °C for later use.

Hydrodynamic radius measurements were conducted with the prepared suspensions as a function of pH. The pH of the suspensions was adjusted with 0.01 mol/L aqueous solutions of hydrochloric acid and sodium hydroxide within a pH range of 2.6 to 9.5. The resulting suspensions were then subjected to DLS analysis to determine the hydrodynamic radius of the nanoparticles, as described in Section 2.5.

### 2.6. Statistical Analysis

The obtained results were statistically analyzed using Statistica software version 7.0 (Statsoft Inc., São Paulo, Brazil) and Tukey’s test with a significance level of 5%.

## 3. Results and Discussion

### 3.1. Characterization: Polymer Precursors and Precipitates of Chitosan/ι-Carrageenan

#### 3.1.1. Metallic Content Within the Commercial Polysaccharides

The polysaccharides, precursors of the coacervates, were degraded by heating at 600 °C. The resulting solid materials were digested in a 5.0% *v*/*v* aqueous nitric acid solution, and the resulting solutions were characterized by FAAS. The concentration of metallic ions (Na^+^, Ca^2+^, and Mg^2+^) in the polysaccharides was determined, and the results are compiled in Table 3. The concentrations of metallic ions in the samples are presented in milligrams per gram of polysaccharide (mg/g).

The as-obtained ι-carrageenan contains 158 mg/g of Ca^2+^, while the purified sample has only 24.85 mg/g. Dialysis effectively removed many Ca^2+^ ions from the commercial ι-carrageenan structure. These Ca^2+^ ions are present in commercial samples due to precipitation processes used in the polysaccharide extraction and neutralization steps, where ι-carrageenan is often extracted with calcium hydroxide [25]. Additionally, Ca^2+^ ions can be incorporated into polysaccharide samples to promote gelation in food industry samples [2]. Ca^2+^ ions act as stabilizing agents for ι-carrageenan, as a single Ca^2+^ ion is required to stabilize two sulfate groups and perform “physical cross-linking” between two adjacent polysaccharide chains, leading to their gelation. Our group previously showed that scarce Ca^2+^ ions were immobilized at the junction zones in 1% ι-carrageenan gel. However, with an increase in the gel concentration to 2%, the proportion of aggregated helices increased, resulting in higher crosslinking density, smaller junction zones, and smaller pore sizes in the calcium phosphate/ι-carrageenan composites [20].

Divalent and monovalent metallic ions stabilize the double helix structures of ι-carrageenan, which results in the gelation of the polymer solution [26]. This explains the high content of metallic ions in the ι-carrageenan structure, including Ca^2+^, Mg^2+^, and Na^+^ (Table 3). The purification of ι-carrageenan through the dialysis was efficient, significantly reducing the concentration of Ca^2+^ ions from 158 mg/g to 24.85 mg/g in the dialyzed polymer. However, there were no significant changes in the concentrations of Mg^2+^ and Na^+^, as these ions remain in the ι-carrageenan structure as counter ions to the sulfate groups, stabilizing them.

A similar result was observed with the chitosan samples. The Ca^2+^ content decreased from 25.7 mg/g to approximately 9 mg/g in the purified chitosan. Mg^2+^ and Na^+^ ions are present in low concentrations in chitosan, and their levels do not show significant variation after purification. Chitosan is primarily obtained from the partial deacetylation reaction of chitin in an alkaline medium, often in NaOH and Ca(OH)_2_ [10,27], which explains the presence of Na^+^ and Ca^2+^ ions. Additionally, Ca^2+^ ions are found in chitosan because calcium is essential for forming crustaceans’ exoskeletons, the primary chitin source. Calcium, magnesium, and sodium are essential minerals, justifying their presence in chitosan [28,29]. Furthermore, amino and hydroxyl groups in chitosan can coordinate with metallic ions [30], accounting for the levels of Ca^2+^, Mg^2+^, and Na^+^ in the purified chitosan (Table 1).

#### 3.1.2. Molecular Weight of the Polysaccharides

The ${\bar{M}}_{w}\;$ of polysaccharides in solution was determined by Static Light Scattering (SLS) measurements. This parameter is essential for the characterization of polymers, as it directly influences their physicochemical properties, such as viscosity, solubility, and stability in aqueous solutions. ${\bar{M}}_{w}\;$ is also related to the ability to form molecular networks and the intermolecular interactions between polymer chains, which are essential for the formation of stable three-dimensional structures [31]. These properties impact the ability of the polymer to form nanoparticles via complexation processes (coacervation). The ${\bar{M}}_{w}s\;$ were determined in duplicate, and their measurements, including the coefficient *A*_2_, are compiled in Table 3.

**Figure 1:**
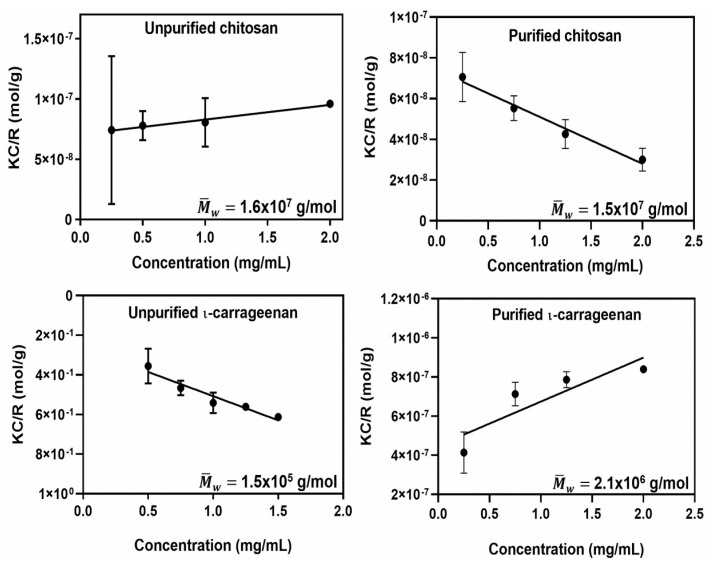
Graph of the *KC*/Δ*R* ratio as a function of the polymer concentration (mg/mL), where Δ*R* is the Rayleigh ratio of a reference solvent, and *K* is the optical constant. The linear coefficient of the curve is related to the ${\bar{M}}_{w}$ of the polysaccharides. The measurements were obtained from duplicates (n = 2).

Figure 1 shows the *KC*/Δ*R* curves as a function of the concentration (mg/mL) of the polymer solutions. In this relationship, K is an optical constant that correlates the refractive index of the solutions with ${\bar{M}}_{w}$, while Δ*R* represents the Rayleigh ratio, a measure of light scattering intensity. The analysis of the *KC*/Δ*R* curves provides simultaneous information about ${\bar{M}}_{w}$ and *A*_2_. The intercept of the curves with the y-axis is used to determine ${\bar{M}}_{w}$, while the slope allows obtaining *A*_2_. This method is effective for verifying how different concentrations affect the behavior in solution, being essential for optimizing processes that depend on the stability and solubility of the polysaccharides. The ${\bar{M}}_{w}$ for the unpurified polymers is 1.5 × 10^5^ for ι-carrageenan and 1.6 × 10^7^ g/mol for chitosan, and ${\bar{M}}_{w}$ for the purified polysaccharides are 2.1 × 10^6^ for ι-carrageenan) and 1.5 × 10^7^ g/mol for chitosan (Table 1).

The increase in the molar mass of carrageenan after dialysis occurs due to the removal of small molecules, such as monomers, oligomers, and other low-molecular-mass impurities, which cross the cellulose membrane with a molecular cut-off weight of 12 kDa. This membrane allows the passage of ι-carrageenan molecules with a mass below 12,000 g/mol, retaining only the larger chains in the sample. With the elimination of these low-molecular-mass fractions, a purified sample is enriched with chains of greater molecular weights, which increase the weighted average molar mass (*M_w_*), dominated by larger molecules. This configuration in the molecular distribution is specific to polymers and polysaccharides, where the purification process results in a higher average molar mass in the final sample [32]. Conversely, there is no significant change in ${\bar{M}}_{w}$ for chitosan after purification.

The ${\bar{M}}_{w}$ of dialyzed chitosan is 1.5 × 10^7^ g/mol, close to the ${\bar{M}}_{w}$ value (1.1 × 10^6^ g/mol) reported in another study [2]. Polysaccharides are polymers with a high degree of polydispersity, resulting in a wide range of reported ${\bar{M}}_{w}$ values. The experimental conditions used in synthesizing chitosan also influence its ${\bar{M}}_{w}$. Chitosan is primarily obtained through the partial deacetylation of chitin in an alkaline medium. The alkaline environment leads to partial hydrolysis of the polysaccharide, reducing the ${\bar{M}}_{w}$ of chitosan compared to chitin [33,34]. The chitin extraction process also impacts the ${\bar{M}}_{w}$, as the extraction time, temperature, and reagent concentrations directly affect it [35]. Typically, chitin is suspended in concentrated NaOH solutions (40–60%) for durations ranging from 0.5 to 24 h at relatively high temperatures (50 to 130 °C) [36]. These conditions promote the partial hydrolysis of the polysaccharide, decreasing the ${\bar{M}}_{w}$.

The purified ι-carrageenan has an ${\bar{M}}_{w}$ of 2.1 × 10^6^ g/mol, which is close to the ${\bar{M}}_{w}$ values commonly reported in the literature, typically ranging from 2.9 × 10^4^ to 9.1 × 10^5^ g/mol [37]. The extraction method of ι-carrageenan is one of the primary factors influencing its ${\bar{M}}_{w}$ [38]. The extraction process involves heating red algae treated with an alkaline solution, such as calcium hydroxide (Ca(OH)_2_), at high temperatures to extract ι-carrageenan. The resulting solution is filtered, and the polymer is precipitated, washed, and dried [39,40]. The high temperature during the extraction process also promotes partial hydrolysis of the polymer, reducing its ${\bar{M}}_{w}$ [41]. Additionally, the properties of ι-carrageenan depend on environmental conditions such as the harvest season [42,43], location and climate, growth conditions (salinity, depth, nutrients), and growth period [44]. These factors explain the ${\bar{M}}_{w}$ variations reported for ι-carrageenan samples.

Negative values for the second virial coefficient (*A*_2_) indicate a predominance of attractive forces among polymer chains in solution. This suggests that polymer–polymer interactions are stronger and more attractive than polymer–solvent interactions compared to ideal gas behavior. A negative *A*_2_ coefficient implies that these attractive polymer–polymer interactions contribute to the non-ideality of the solution. Conversely, a positive *A*_2_ coefficient in polymer solutions indicates stronger polymer–solvent interactions than polymer–polymer interactions. In this case, the polymer chains disperse in the solvent rather than aggregate. The significant polymer–solvent interactions lead to a uniform dispersion of the polymer chains and contribute to the stability of the solution [45].

Negative *A*_2_ values explain the decreasing *KC*/Δ*R* ratio as polymer concentration increases. The unpurified ι-carrageenan and purified chitosan exhibit negative *A*_2_ coefficients (Table 3), indicating that attractive forces prevail among polymer chains in solution, favoring polymer aggregation. The high content of Ca^2+^ in ι-carrageenan (unpurified sample) stabilizes the ionized sulfate groups, promoting polymer chain coiling (favoring aggregation) and the occurrence of attractive interactions between unpurified ι-carrageenan chains.

Dialysis impacts chitosan properties differently than ι-carrageenan, as commercial chitosan is not acquired in a salt form. Dialysis removes charged species (ions) from chitosan [46], making amino groups more accessible to interact on adjacent polymer chains in the purified chitosan. Therefore, eliminating ionic impurities decreases chitosan’s affinity for water molecules, leading to a negative *A*_2_ coefficient. This indicates more significant polymer–polymer interactions relative to polymer–solvent interactions.

#### 3.1.3. Infrared Spectroscopy

**Figure 2:**
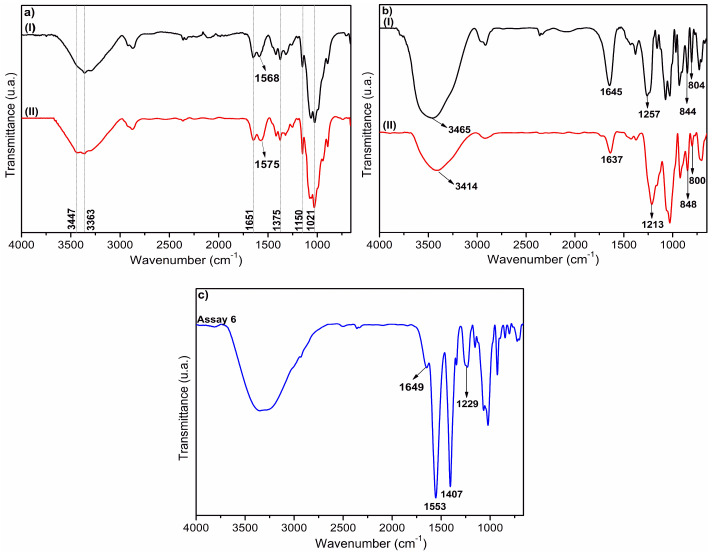
FTIR spectra: (I) Unpurified chitosan and (II) purified chitosan (**a**), (I) unpurified ι-carrageenan and (II) purified ι-carrageenan (**b**), and precipitate obtained from assay 6 (**c**). Experimental conditions of assay 6: pH 4.4, purified ι-carrageenan at 0.3 mg/mL, purified chitosan at 0.3 mg/mL, polycation/polyanion volume ratio of 2, and mass ratio of 66.6/33.4.

Figure 2a presents the FTIR-ATR spectra of chitosan. Both spectra before and after purification exhibit similar bands, which is characteristic of the chemical structure of this polysaccharide. The profiles are similar in terms of band intensity and wavenumber assignment. The stretching (O–H and N–H) occurs at 3447 and 3363 cm^−1^. The band at 1651 cm^−1^ is attributed to the asymmetric stretching of the N–C=O bonds of amide I, deriving from the acetylated units of chitin that remain in chitosan. The band at 1568 cm^−1^ (FTIR-ATR spectrum (I) for unpurified chitosan) and 1575 cm^−1^ (FTIR-ATR spectrum (II) for purified chitosan) results from the bending deformation of the N-H bonds combined with the stretching of the C–N bond (Figure 2a). The band at 1375 cm^−1^ is attributed to the angular deformation of the C-H bonds in the methyl groups of the acetamide moieties. In the range between 1150 and 1021 cm^−1^, stretching of the C–O bonds of primary alcohols and C-O-C bonds of ether groups are observed [47,48,49].

The FTIR-ATR spectrum of unpurified ι-carrageenan reveals bands associated with the stretching of O-H bonds at 3465 cm^−1^, angular deformation (bending) of water molecules at 1645 cm^−1^, symmetric stretching of O=S=O bonds on sulfate groups at 1257 cm^−1^, and stretching (symmetric and asymmetric) of sulfate groups (–O–SO_3_) in the D-galactose-4-sulfate and D-galactose-2-sulfate repeat units at 844 and 804 cm^−1^, respectively (Figure 2b(I)) [50,51]. For purified ι-carrageenan, the bands attributed to the stretching of O–H bonds occur at 3414 cm^−1^, angular deformation (bending) of water at 1637 cm^−1^, symmetric stretching of O=S=O bonds on sulfate groups at 1213 cm^−1^, and stretching of the sulfate groups (–O–SO_3_) in the D-galactose-4-sulfate and D-galactose-2-sulfate units at 848 and 800 cm^−1^, respectively (Figure 2b) [51].

On the other hand, there is a significant alteration in the profile of the ι-carrageenan spectrum after purification (Figure 2b(II)). The main changes occur in the bands associated with the symmetric stretching of the O=S=O bonds, O–H stretching, and water deformation. Shifts and changes in intensities related to these bands are observed. For example, the O–H stretching and the water deformation bands decrease significantly in intensity in the FTIR-ATR spectrum of purified ι-carrageenan compared to the bands in the spectrum of the unpurified sample. These bands shift from 3465 and 1645 (FTIR-ATR spectrum of the unpurified ι-carrageenan) to 3414 and 1637 cm^−1^ (FTIR-ATR spectrum of the purified sample). Dialysis reduces the water content of ι-carrageenan. The high quantity of metallic ions is suggested to favor a high water content in the unpurified ι-carrageenan. Dialysis removes the excess ions, and lyophilization contributes to the removal of hydration water in the purified ι-carrageenan due to its lower content of metallic ions. Additionally, the band attributed to the O=S=O bonds enlarges in the spectrum of purified ι-carrageenan and shifts from 1257 cm^−1^ (FTIR-ATR spectrum of the unpurified sample) to 1213 cm^−1^ (FTIR-ATR spectrum of purified ι-carrageenan). This occurs due to the reorganization of ι-carrageenan chains in the purified material, resulting from removing a large quantity of Ca^2+^ ions.

These results indicate that the purification process modifies the chemical composition of ι-carrageenan, corroborating the FAAS and DLS results. There are no significant differences between the FTIR-ATR spectra of chitosan before and after purification since chitosan is not commercialized in the salt form like ι-carrageenan, and the impurity concentration in chitosan is lower compared to the impurity concentration in ι-carrageenan. This explains the differences in the FTIR-ATR spectra of the polymer precursors.

Figure 2c shows the FTIR-ATR spectrum of the precipitate obtained from assay 6 (Table 1). In this spectrum, the band attributed to the asymmetric stretching of the C=O bonds of amide I occurs at 1649 cm^−1^. The intense band at 1553 cm^−1^ is associated with the combined bending deformation of ^+^N–H bonds and C–N^+^ stretching on protonated chitosan chains [52]. Another intense band at 1407 cm^−1^ is likely attributed to the angular deformation of the C–H bonds in methyl groups of acetamide moieties. Additionally, a reduction in the band’s intensity at 1229 cm^−1^, attributed to the sulfate group in the ι-carrageenan chains, is observed. These results indicate the formation of polyelectrolyte complexes as a precipitate [53,54].

#### 3.1.4. Thermal Analysis

**Figure 3:**
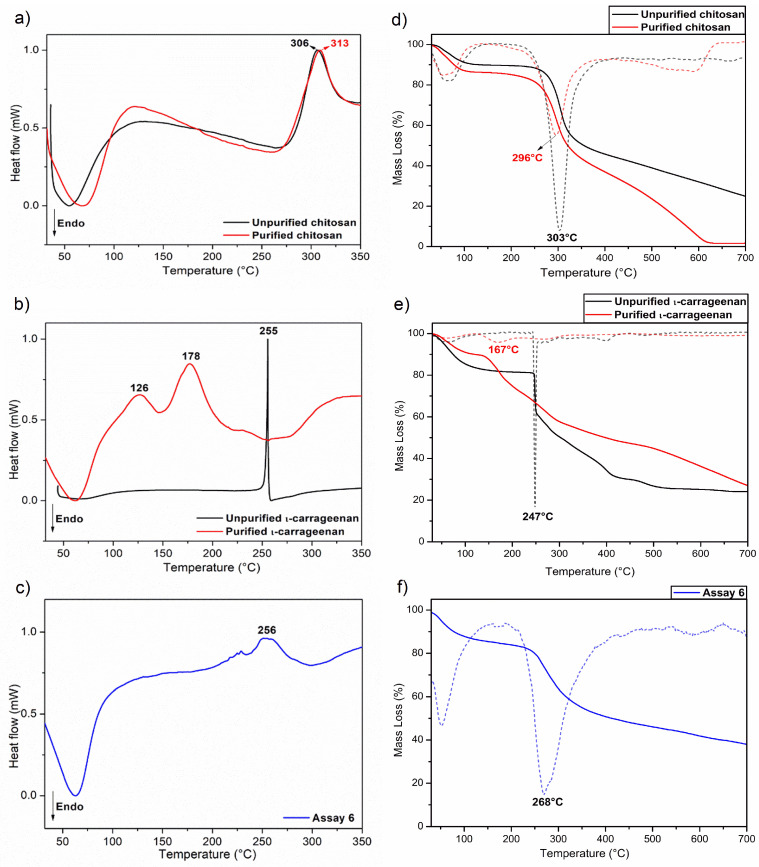
DSC curves: Chitosan (**a**), ι-carrageenan (**b**), and ι-carrageenan/chitosan precipitate obtained from assay 6 (**c**). TGA curves: chitosan (**d**), ι-carrageenan (**e**), and ι-carrageenan/chitosan precipitate obtained from assay 6 (**f**). The dashed lines in (**d**–**f**) represent the first derivative of the TG curves. Experimental conditions of assay 6: pH 4.4, purified ι-carrageenan at 0.3 mg/mL, purified chitosan at 0.3 mg/mL, polycation/polyanion volume ratio of 2, and mass ratio of 66.6/33.4.

Figure 3a–c shows the DSC curves of the polymers before and after purification by dialysis and the precipitate of ι-carrageenan/chitosan obtained from assay 6 (Table 1). The DSC curves of the polymers reveal distinct thermal events. The first event is endothermic, appearing in the range of 30 °C to 110 °C and corresponding to the samples’ dehydration. The second event is exothermic and occurs above 300 °C in the DSC curves for chitosan (Figure 3a). However, the exothermic event in the DSC curves of ι-carrageenan happens at 255 °C for the unpurified sample and at 126 °C and 178 °C for the purified polysaccharide (Figure 3b). These exothermic events are attributed to the degradation of the polymer chains, primarily related to the cleavage of glycosidic bonds [55].

Like the FTIR spectrum profiles, dialysis has a more significant influence on the DSC curves for ι-carrageenan than for chitosan DCS curves. Metallic cations stabilize the double-helix configurations of ι-carrageenan [56]. However, the unpurified sample contains a very high amount of Ca^2+^ ions, resulting in the early degradation of unpurified ι-carrageenan (255 °C) compared to the more pronounced exothermic peak at 178 °C in the DSC curve of purified ι-carrageenan. The purified ι-carrageenan also includes metallic ions, which stabilize the polysaccharide chains. Additionally, dialysis removes oligomers of ι-carrageenan, likely intensifying the interactions between the ι-carrageenan chains, as the purified sample has a higher ${\bar{M}}_{w}$ than the unpurified one. This increases the thermal stability of the purified material, explaining the DSC curve profiles of ι-carrageenan (Figure 3b).

The exothermic peak attributed to degradation is narrower in the DSC curve of unpurified ι-carrageenan compared to the exothermic peaks in the DSC curve of the purified sample (Figure 3b). This is likely due to the occurrence of ionic pairs in the structure of commercial ι-carrageenan. These results are consistent with the FAAS data, which indicate a high content of metallic ions in the unpurified ι-carrageenan, and they agree with other data reported in the literature, which show the effect of ionic pairs in the characterization of polysaccharides [46].

The DSC curve profile of the ι-carrageenan/chitosan precipitate obtained from assay 6 (Figure 3c) shows degradation events, with the most pronounced at 256 °C. This temperature is intermediate compared to the degradation temperatures of the dialyzed precursor polymers. These results also suggest the formation of the ι-carrageenan/chitosan polyelectrolyte complex, as the DSC curve profile of the precipitate differs from the precursors’ DSC curve profiles [57]. ι-Carrageenan interacts with chitosan mainly through electrostatic interactions. The FTIR results indicate that the protonated amino groups (pK_a_ = 6.5) of chitosan and the sulfate groups (pK_a_ = 2.6) [58] in ι-carrageenan are interacting in the polyelectrolyte complex obtained as a precipitate at pH 4.4 (assay 6). This pH condition favors the partial ionization of both polyelectrolytes and the formation of the polyelectrolyte complex.

Figure 3d–f show the thermogravimetric curves of the polysaccharides before and after purification. The first thermal event occurs in the range of 25 °C to 150 °C for the chitosan and ι-carrageenan samples, with the mass change attributed to the removal of water and volatile components from the polymer structures [59]. In this first event, the relative mass change percentages are 8.69% and 12.5% for the unpurified and purified chitosan samples, respectively, and 19% and 12% for the unpurified and purified ι-carrageenan samples. For chitosan (Figure 3d), the main thermal event occurs between 296 °C and 303 °C, related to polymer degradation, with mass changes of 87.59% and 82.0% for the unpurified and purified samples, respectively [60,61]. For ι-carrageenan (Figure 3e), the second thermal event occurs around 247 °C for unpurified ι-carrageenan and 167 °C for purified ι-carrageenan, associated with the degradation of the polymer backbone via glycosidic bond cleavage [62]. The purified ι-carrageenan sample shows different thermal stability compared to the unpurified one, possibly due to a lower concentration of counterions in its composition, as indicated by the FAAS analysis.

Figure 3f presents the thermogram for the ι-carrageenan/chitosan precipitate obtained in experiment 6. It shows the first thermal event related to the change of water and volatile components, with a relative mass alteration of 12.13%. The second thermal event occurs around 268 °C, an intermediate degradation temperature compared to those of its respective precursors. This change in decomposition temperature may be associated with intermolecular forces between the chitosan and ι-carrageenan polymer chains, suggesting the formation of a polyelectrolyte complex and corroborating the thermal events observed in the DSC curves.

### 3.2. Characterization of the Nanoparticles Obtained in Suspension

#### 3.2.1. Hydrodynamic Radii and Zeta Potential Measurements

Only purified polysaccharides were used in the preparation of the coacervates. Measurements of hydrodynamic radius, zeta potential (ζ), and polydispersity index (PDI) of nanoparticles in suspension at 25 °C and 37 °C are provided in Table 4 and Table 5. The preparation of nanoparticles considered various experimental variables, including the concentration of polyelectrolytes, pH, order of addition of polyelectrolytes, and polymer mixture ratio.

**Figure 4:**
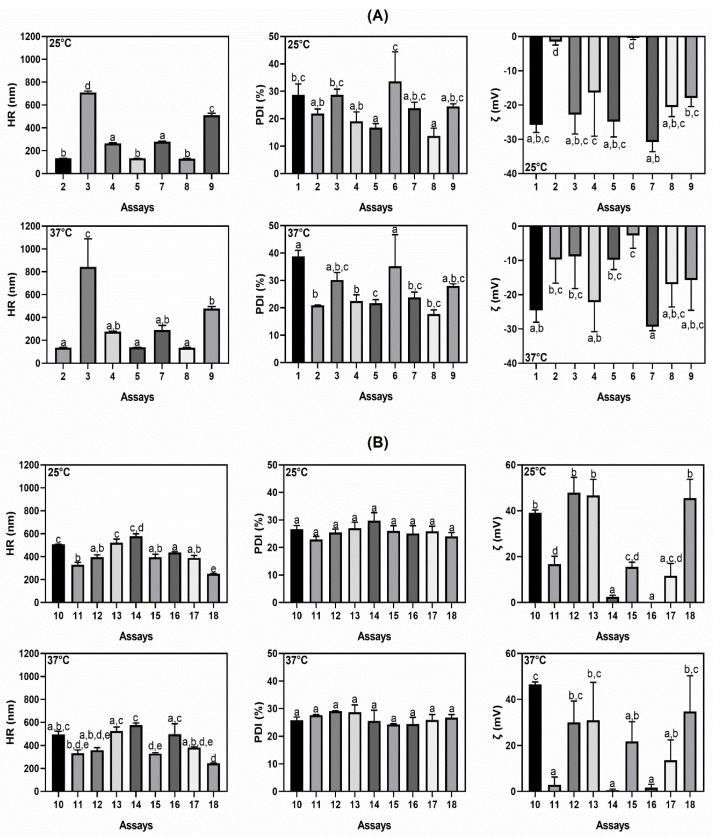
Comparison of DLS results: Measurements of the hydrodynamic radius (HR) or average wet diameter of particles in suspension, zeta potential (ζ, mV) of particles in suspension, and polydispersity index (PDI, %) of particles in suspension. Measurements were taken at 25 °C and 37 °C with three replicates (n = 3). (**A**) represents samples with excess ι-carrageenan (Table 4), and (**B**) shows samples with excess chitosan (Table 5). Different lower-case letters in Figure 4 indicate significant differences between the measurements (*p* ≤ 0.05).

Figure 4 compares bar graphs of the DLS measurements at 25 °C and 37 °C performed with the coacervate suspensions described in Table 4 and Table 5. Figure 4A displays the results for the coacervates obtained with higher ι-carrageenan concentration (experiments 2, 3, 4, 5, 7, 8, and 9) than chitosan. In contrast, Figure 4B shows the results for the samples prepared from more concentrated chitosan solutions than ι-carrageenan (experiments 10 to 18).

The average size of the particles created with higher ι-carrageenan concentrations (pH 3.6) than chitosan concentration ranges from 131 to 511 nm. At pH 4.0, the average particle diameter is between 278 and 290 nm, while at pH 4.4, the nanoparticles exhibit average diameters ranging from 265 to 9811 nm (Table 4). Coacervates prepared with higher chitosan concentrations (pH 3.6) than ι-carrageenan exhibit average sizes ranging from 246 to 578 nm. At pH 4.0, the average particle size is between 330 and 394 nm, while at pH 4.4, the coacervates have average diameters in the range of 359 to 523 nm (Table 5). These results include diameter measurements taken at temperatures of 25 and 37 °C.

The ζ values of particles obtained with an excess of ι-carrageenan are in the ranges of −2 to −25 mV (pH 3.6), −29 to −31 mV (pH 4.0), and −2 to −26 mV (pH 4.4) (Table 4). Nanoparticles obtained with an excess of chitosan have ζ values in the ranges of +1 to +45 mV (pH 3.6), +16 to +22 mV (pH 4.0), and 0 to +48 mV (pH 4.4) (Table 5). As expected, an excess of chitosan promotes coacervates with positive charge density, while an excess of ι-carrageenan results in particles with negative charge density.

Nanoparticles with an excess of polyanion obtained by inverting an aliquot of 24 mL of ι-carrageenan solution (0.9 mg/mL) into a chitosan solution at 0.3 mg/mL (12 mL) (experiment 8, Table 5) result in nanoparticles with the smallest average diameter (131 ± 2 nm at 25 °C and 135 ± 4 nm at 37 °C) and ζ of −21 ± 2 mV at 25 °C and −17 ± 7 mV at 37 °C. This result was achieved under pH 3.6 conditions. However, when only the pH condition is altered, when the pH increases from 3.6 to 4.4, a significant increase in the average particle size is observed, with aggregation resulting in a diameter of 4547 nm (assay 1, Table 4). The ζ value is more negative, indicating −26 ± 2 mV and −25 ± 3 mV at 25 °C and 37 °C, respectively.

The increase in pH favors the deprotonation of chitosan and an increase in the negative charge density in the coacervates, explaining the more negative ζ values in assay 1 compared to assay 8 (Table 4 and Figure 4A). The increase in pH favors the formation of aggregates due to the partial deprotonation of chitosan. This favors the interaction (hydrogen bonding) between deprotonated chitosan chains, resulting in aggregated particles with high PDI (Table 4 and Figure 4A).

Results similar to experiment 8 were achieved in experiment 5, when the volume/volume ratio of polyanion to polycation was increased from 2 to 8 at pH 3.6 (Table 4). However, the aggregation of the coacervates was significantly reduced when the pH was increased to 4.4 (assay 4), obtaining particles of 265 ± 6 nm (PDI = 19 ± 3% and ζ = −16 ± 1 mV) and 277 ± 3 nm (PDI = 23± 2% and ζ = −22 ± 9) at 25 °C and 37 °C, respectively. This effect is due to the increased volume/volume ratio of polyanion to polycation, minimizing the partial deprotonation of the chitosan chains, reducing the intensity of intermolecular interactions between the polymer chains, and suppressing particle aggregation (Table 4 and Figure 4A).

A concentration of ι-carrageenan of 0.3 mg/mL and a fixed volume/volume ratio of polyanion/polyanion at 8 resulted in nanoparticles with average sizes and ζ values of 132 ± 1 nm and −2 ± 1 mV (25 °C) and 137 ± 3 nm and −10 ± 7 mV (37 °C) at pH 3.6 (assay 2, Table 4, Figure 4A). Once again, the average particle diameter increases when the pH is increased to 4.4. However, the average diameter (assay 3) increases less compared to the increase seen in assay 1 (Table 4 and Figure 4A). This is due to the lower concentration of ι-carrageenan, 0.3 mg/mL in assays 2 and 3, versus 0.9 mg/mL in assay 1.

Effects similar to those observed in experiments 1, 4, 5, and 8 were noticed in experiments 6 and 7. However, experiment 7 produced particles (278 ± 5 nm) with the smallest ζ value, reaching −31 ± 3 mV at 25 °C. These values fluctuate slightly in measurements taken at 37 °C, indicating ζ of −29 ± 1 mV and a diameter of 290 ± 41 nm. These results are achieved when the pH is fixed at 4.0, with a polyanion/polyanion ratio 5 (assay 7, Table 4). Particles with low PDI were obtained by adjusting experimental factors such as pH, concentration of ι-carrageenan, mixing order, and the volume/volume ratio of polyanion solution to polycation solution at 0.3 mg/mL. A ζ value of −30 mV or lower is required for coacervates with negative charge density and high stability in suspension. This result was achieved in experiment 7 (Table 4, Figure 4A).

The results are entirely different when the mixing order is reversed, meaning when chitosan solutions are poured into the 0.3 mg/mL ι-carrageenan solution. Reversing the mixing order also means reversing the volume/volume ratio of the solutions in the final mixture of polyelectrolytes. Particles with excess chitosan exhibit average diameters in suspension ranging from 246 to 578 nm (pH 3.6), 330 to 394 nm (pH 4.0), and 359 to 523 nm (pH 4.4) (Table 5 and Figure 4B). With the reversed addition order, the average diameter of the coacervates increases. For example, experiment 8 results in particles with an average diameter of 131 ± 2 nm (25 °C) and 135 ± 4 nm (37 °C); however, when the chitosan solution is poured into the ι-carrageenan solution, the average diameter and ζ increase to 389 ± 22 nm (25 °C) and 382 ± 8 nm (37 °C) with ζ +12 ± 5 mV (25 °C) and +14 ± 9 mV (37 °C) (experiment 17, Table 5 and Figure 4B). As expected, higher chitosan concentration than polyanion content promotes particles with positive ζ (Table 5 and Figure 4B).

The smallest particle diameter of the samples with excess polycation was obtained from experiment 18, indicating 252 ± 1 nm (25 °C) and 246 ± 2 nm (37 °C) with ζ +45 ± 8 mV and ζ of +35 ± 8 mV, respectively. Experiment 18 was the condition that provided the best properties for the chitosan-excess particles, including smaller average diameter and higher ζ, above +30 mV (Table 5).

As with the coacervates prepared with excess ι-carrageenan, the best results for particles with excess chitosan are achieved at pH 3.6. However, this result is obtained with a ratio of 2 (polycation/polyanion) and solutions of ι-carrageenan and chitosan at 0.3 mg/mL (assay 18, Table 5). A pH of 3.6 favors the protonation of chitosan, promoting high and positive ζ, which provides stability to the suspension due to electrostatic repulsion between the particles [63]. When the pH increases from 3.6 (assay 18) to 4.4 (assay 16), keeping the other conditions fixed (concentrations of the solutions, polycation/polyanion ratio), the average diameter of the particles significantly increases to 437 ± 6 (25 °C) and 500 ± 80 (37 °C) with ζ values of 0 and +2 ± 1 mV, respectively. The increase in pH partially deprotonates the chitosan chains, significantly reducing the ζ. Similar ζ results occur in experiments 2, 6, 14, and 16. The ζ is close to zero for all coacervates obtained under these conditions. These conditions likely promote a situation close to the equivalence between polycations and polyanions, promoting a net charge close to zero. Therefore, the resulting material was obtained as a precipitate [64].

**Scheme 1:**
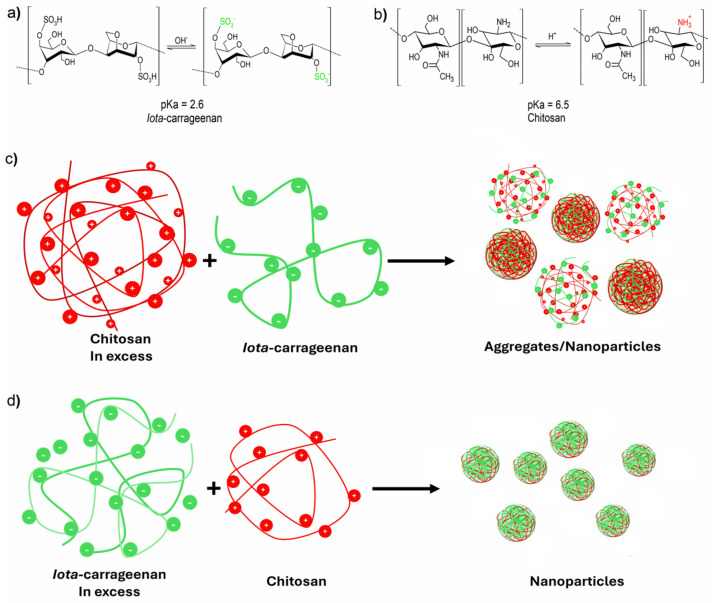
The chemical structures of ι-carrageenan and chitosan under equilibrium conditions in aqueous solutions are shown (**a**,**b**). Aggregated coacervates are formed when an excess of chitosan is used due to the partial deprotonation of chitosan chains (**c**). Conversely, smaller coacervates are obtained when an excess of ι-carrageenan is present during preparation as a result of the partial deprotonation of the sulfate groups in ι-carrageenan (**d**).

Scheme 1 depicts the chemical structures of the polysaccharides ι-carrageenan and chitosan, highlighting the species that coexist in equilibrium within aqueous solutions of these polysaccharides (Scheme 1a,b). Scheme 1c illustrates the effect of increasing particle size when prepared with higher concentrations of chitosan. As the pH increases, the chitosan chains become partially deprotonated, leading to a higher −NH_2_/-NH_3_^+^ ratio. This intensifies intermolecular hydrogen bonding interactions, promoting particle aggregation (Scheme 1c). An opposite effect is observed when the concentration of polyanion is higher. In this case, the increase in pH induces partial deprotonation of the ι-carrageenan chains. This enhances the negative charge density on the particles, increasing their stability and preventing aggregation (Scheme 1d).

Coacervate suspensions with hydrodynamic radii or wetted average diameters close to 200 nm showed lower PDI values. These experiments include assays 4, 5, and 8, with PDI values of 19 ± 3% (25 °C) and 23 ± 2% (37 °C), 17 ± 1% (25 °C) and 22 ± 1% (37 °C), and 14 ± 3% (25 °C) and 18 ± 2% (37 °C), respectively (Table 1 and Table 2 and Figure 4). Low PDI values (around or close to 10%) indicate suspensions with monodisperse colloidal particles. The common factor among these experiments is the excess of ι-carrageenan, and assays 5 and 8 were performed at pH 3.6. The PDI values of the particles obtained in the other experiments were below 30% (except for assay 1). It is worth noting that PDI values higher than 30% indicate particles with a wide size distribution, indicating high heterogeneity [65].

The DLS results indicate that pH is the most crucial factor to be controlled to obtain particles with diameters below 300 nm with ζ values below −30 mV or above +30 mV and PDI close to 10%. pH 3.6 and 4.0 are the best conditions and lead to particles with smaller average diameters compared to pH 4.4. Both chitosan (pK_a_ 6.5) and ι-carrageenan (pK_a_ = 2.6) are partially ionized in the pH range studied, between 3.6 and 4.4. However, the pH 4.4 condition substantially favors the deprotonation of -NH_3_^+^ groups in chitosan, impacting the properties of the coacervates in suspension and favoring particle aggregation. The best pH conditions (3.6 and 4.0) fall within an intermediate range relative to the pK_a_ values of the polyelectrolytes because these pH conditions should promote appropriate amounts of cationic and anionic sites, which stabilize each other through Coulombic interactions, and the excess of polyanion or polycation generates suspension stability. The excess of one of the polyelectrolytes in the composition of the coacervates is of utmost importance, as it results in ζ values falling within the stability range (≤−30 mV or ≥+30 mV) [66].

The volume/volume ratio of the polymers and the mixing order significantly influence the values of hydrodynamic radius and PDI of the particles in suspension. In general, the excess of chitosan and the inversion of chitosan in ι-carrageenan solutions result in particles with larger diameters than those produced with an excess of ι-carrageenan and the inverted addition order of the solutions. These results are in accordance with other reported data [67]. This phenomenon can be explained by the high capacity of chitosan to form aggregates, whereas its solubilization depends mainly on the degree of acetylation and pH [68]. The partial deprotonation of -NH_3_^+^ groups increases the concentration of amino groups (-NH_2_). The -NH_2_ groups and hydroxyl groups interact with each other through hydrogen bonding, favoring particle aggregation [69,70].

The molecular weight of the polymers should also influence the properties of the particles [71,72]. Increasing the polyelectrolytes’ molecular weight increases the particles’ average size in suspension [73,74]. This study also observed this trend since chitosan has a higher molecular weight than ι-carrageenan. Therefore, an excess of chitosan contributes to an increase in the average diameter of the coacervates.

DLS measurements were performed at two temperatures, 25 °C and 37 °C. Temperature does not significantly affect the average size of the nanoparticles in suspension, but it does influence the ζ measurements, especially in experiments 2, 3, 5, 10, 11, 12, 13, 15, and 18. ζ values seem to decrease with increasing temperature. This phenomenon depends on the colloidal properties and characteristics of the dispersing medium, such as the electrical double layer’s electrostatic property and the medium’s electrophoretic capacity. An increase in temperature reduces both the solvent viscosity and the electrical permittivity of the medium. Temperature changes affect the properties of colloidal particles, influencing the surface charge density [75].

#### 3.2.2. Hydrodynamic Radii as a Function of pH

To evaluate the influence of pH on the hydrodynamic radius of the nanoparticles, experimental condition 7 was selected (experimental Section 2.4). However, for this specific study, instead of obtaining nanoparticles in a buffer solution (pH 4.0), they were prepared from aqueous polysaccharide solutions at pH 3.6. The pH of these aqueous solutions was adjusted with acetic acid, allowing for easy pH variation in the hydrodynamic radius measurements as a function of pH (Figure S1, Supplementary Materials).

The hydrodynamic radius obtained with the particles obtained from experiment 7 (pH 4.0, Table 4) was 278 nm, while the hydrodynamic radius obtained from mixing aqueous solutions (pH 3.6) was 245 nm. In the previous section, we discussed the influence of pH on the properties of particles in suspension. This effect is related to the deprotonation of chitosan and ι-carrageenan chains, which is favored as the pH increases.

Hydrodynamic radius measurements as a function of pH show a variation in the hydrodynamic radius (Figure S1). The nanoparticles are pH-responsive, with a smaller hydrodynamic radius in suspensions with pH values between 2.6 and 4.0 but increasing from 273 nm (pH 3.1) to 415 nm (pH 7.0). This behavior is mainly due to the deprotonation of the ι-carrageenan chains (pK_a_ 2.6) as the pH increases. In contrast, the nanoparticles contain a higher concentration of ι-carrageenan relative to chitosan. The deprotonation of the ι-carrageenan chains increases interaction with water molecules, causing the suspension particles to swell, consequently increasing the hydrodynamic radius [75].

#### 3.2.3. Scanning Electron Microscopy of the Nanoparticles

**Figure 5:**
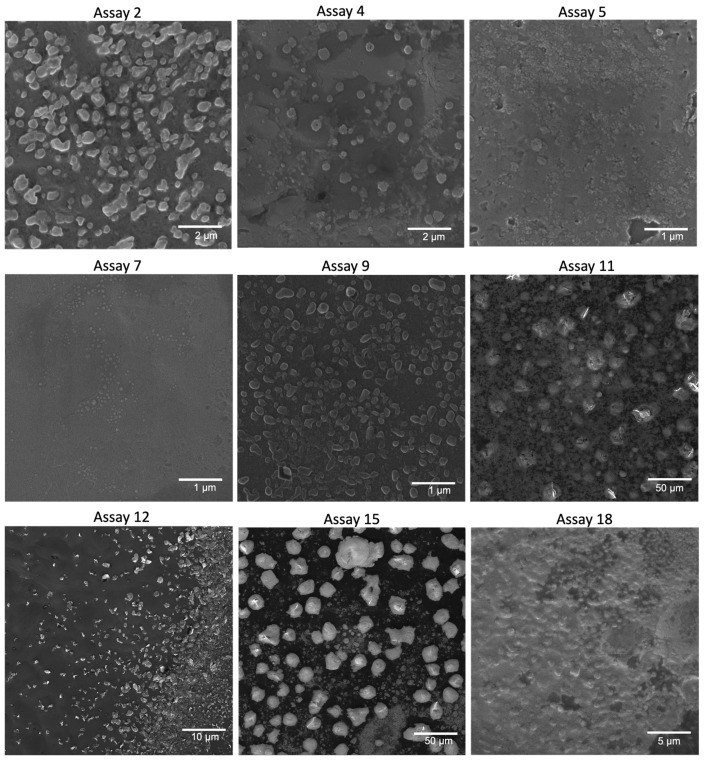
SEM images of the particles. The images corresponding to assays 2, 4, 5, 7, and 9 depict coacervates prepared with an excess of ι-carrageenan (Table 1 and Table 4), while the images corresponding to assays 11, 12, 15, and 18 depict coacervates obtained with an excess of chitosan (Table 2 and Table 5).

Figure 5 presents SEM images of the nanoparticles. Overall, a distribution of particles with spherical geometry is observed. Size distribution curves are presented in Figure S2 (Supplementary Materials). Some samples do not exhibit a well-defined spherical geometry, and the average diameter was determined by considering the furthest points of the particles from each other. The average diameter was obtained from 30 measurements, considering particles randomly selected from the SEM images. The size distribution curves indicate the average diameter values (Figure S2). Table 6 compares the average particle diameters (nm) obtained through DLS measurements and SEM images.

The SEM images of the particles with excess ι-carrageenan (experiments 2, 4, 5, 7, and 9) show homogeneous nanomaterials with a dry diameter range between 44 and 215 nm. It is worth highlighting the SEM image of the nanoparticles from experiment 7, which have a dry average diameter of only 45 ± 11 nm and are dispersed on polystyrene, the substrate used for coacervate deposition. For better visualization, the SEM image of the samples obtained from experiment 7 is presented in the supplementary material with higher magnification (Figure S3). Generally, nanoparticles with excess ι-carrageenan are slightly aggregated compared to particles with excess chitosan. The SEM images of the samples with excess chitosan display highly aggregated particles with spherical geometry and a dry average diameter range between 0.366 μm (366 nm) and 13.5 μm (13.5 × 10^3^ nm) (experiments 11, 12, 15, and 18). The drying process of the suspended particles may influence morphology and dry diameter results, resulting in cluster formation.

The difference in average size between measurements taken with DLS and SEM is notable. This was expected, especially for particles with high ι-carrageenan concentrations (experiments 4, 5, 7, and 9). For example, in experiment 7, the average diameter determined by DLS is 278 ± 5 nm, while the average dry diameter obtained by SEM is 45 ± 11 nm (Table 6). This occurs because DLS measures the hydrodynamic radius of particles in suspension, considering their Brownian motion. At the same time, swelling and hydration contribute to an increase in the average size of the particles [76].

On the other hand, SEM images are captured with the nanoparticles in a dry state, in a static mode on the surface of polystyrene films. Drying contracts the polymeric matrix, reducing the average size of the particles. These results are consistent with findings from DLS and SEM studies of chitosan, hypromellose phthalate, and hyaluronic acid coacervates reported in the literature [77].

However, in experiment 2, the dry particle size (215 ± 61 nm) is larger than the suspended particle size (132 ± 1 nm). This possibly occurs due to the low stability of the coacervate suspension in experiment 2, indicated by a ζ close to zero (−2 ± 1 mV), suggesting aggregation during the drying process before SEM imaging.

On the other hand, SEM images reveal microparticles in experiments 11, 15, and 18 for samples with an excess of chitosan. The average dry particle size is 6.5 × 10^3^ ± 1.5 × 10^3^ nm (experiment 11), 13.5 × 10^3^ ± 2.7 × 10^3^ nm (experiment 15), and 366 ± 93 nm (experiment 18). In these cases, there is a significant increase in the average particle diameter compared to measurements made by DLS. For example, particles provided from experiments 12 and 18 have wet average diameters of 396 ± 20 nm (ζ = +48 mV at 25 °C) and 252 ± 1 nm (ζ = +45 mV at 25 °C), respectively. However, the average dry diameters of these particles determined by SEM images are 620 ± 172 nm (assay 12) and 366 ± 93 nm (assay 18) (Table 6). Zeta potentials of around +45 mV were not sufficient to prevent particle aggregation.

The higher propensity for aggregation of particles with an excess of chitosan than particles with an excess of ι-carrageenan had already been observed in DLS measurements. Chitosan is insoluble in water, and unprotonated amino groups’ presence in its structure leads to aggregate formation. Drying of the particles significantly contributes to this aggregation, resulting in larger diameters of dry particles and the formation of clusters (Table 6).

## 4. Conclusions

The production of coacervates based on dialyzed ι-carrageenan and dialyzed chitosan with average diameters in the nanoscale and stability requires precise control of several factors, including pH, concentration, volume of the polyelectrolyte solutions mixed, and order of addition. Coacervates with dry average diameters of 45 nm and wet average diameters (hydrodynamic radius) of 278 nm (experiment 7) were obtained with an excess of ι-carrageenan, specifically when the ι-carrageenan solution (30 mL at 0.6 mg/mL and pH 4.0) is poured into the chitosan solution (6.0 mL at 0.3 mg/L and pH 4.0). Experiment 7 resulted in stable particles with high polyanion concentration, with a zeta potential of −31 mV at 25 °C. On the other hand, particles with excess chitosan showed no stability and were easily aggregated. Particle aggregation is favored by the increase in pH due to the deprotonation of the amino groups in chitosan. Deprotonation promotes intermolecular interactions, such as hydrogen bonding between the polymer chains, leading to aggregation. Therefore, coacervates with excess chitosan exhibited dry diameters in the micrometer scale. The factors of polyelectrolyte addition order, pH, volume/volume ratio of solutions, and polymer molecular weight significantly influence the formation and properties of the coacervates. This study contributes to a better understanding of the factors governing the production of stable physical nanoparticles in aqueous suspension.

## Figures and Tables

**Table 1:** Experimental conditions used to prepare nanoparticles with an excess of polyanion, dialyzed ι-carrageenan.

Assays	pH	DCA * (mg/mL)	DCH (mg/mL)	PA/PC (*v*/*v*)	PA/PC (*w*/*w*)
1	4.4	0.9	0.3	2	85.7/14.3
2	3.6	0.3	0.3	8	88.8/11.2
3	4.4	0.3	0.3	8	88.8/11.2
4	4.4	0.9	0.3	8	96/4
5	3.6	0.9	0.3	8	96/4
6	4.4	0.3	0.3	2	66.6/33.4
7	4.0	0.6	0.3	5	90.9/9.1
8	3.6	0.9	0.3	2	85.7/14.3
9	3.6	0.3	0.3	2	66.6/33.4

* DCA: Dialyzed ι-carrageenan, DCH: dialyzed chitosan, PA/PC: polyanion/polycation ratio.

**Table 2:** Experimental conditions used to prepare nanoparticles with an excess of polycation, dialyzed chitosan.

Assays	pH	DCH * (mg/mL)	DCA (mg/mL)	PC/PA (*v*/*v*)	PC/PA (*w*/*w*)
10	4.4	0.9	0.3	2	85.7/14.3
11	3.6	0.3	0.3	8	88.8/11.2
12	4.4	0.3	0.3	8	88.8/11.2
13	4.4	0.9	0.3	8	96/4
14	3.6	0.9	0.3	8	96/4
15	4.0	0.6	0.3	5	90.9/9.1
16	4.4	0.3	0.3	2	66.6/33.4
17	3.6	0.9	0.3	2	85.7/14.3
18	3.6	0.3	0.3	2	66.6/33.4

* DCH: dialyzed chitosan, DCA: dialyzed ι-carrageenan, PC/PA: polycation/polyanion ratio.

**Table 3:** Metallic ion content (determined by FAAS), molecular mass (${\bar{M}}_{w}$), and virial coefficient (*A*_2_) (determined through static light scattering (SLS) measurements) of the polymer solutions.

Polysaccharide	Na^+^ (mg/g)	Ca^2+^ (mg/g)	Mg^2+^ (mg/g)	${\bar{M}}_{w}$ (g/mol)	*A*_2_ (mol·mL/g^2^)
Unpurified ι-carrageenan **	2.69	158.22	0.566	1.5 × 10^5^	−6.56 × 10^−5^
Purified ι-carrageenan *	2.23	24.85	0.500	2.1 × 10^6^	1.22 × 10^−4^
Unpurified chitosan **	2.44	25.47	1.75	1.6 × 10^7^	5.74 × 10^−6^
Purified chitosan *	2.47	9.09	0.64	1.5 × 10^7^	−9.93 × 10^−6^

** As-received samples (unpurified) and * purified samples by dialysis.

**Table 4:** DLS measurements performed with suspensions (coacervates) obtained with higher polyanion (ι-carrageenan) concentration concerning chitosan.

				25 °C			37 °C		
Assays	pH	DCA (mg/mL)	PA/PC *	HR (nm)	ζ (mV)	PDI (%)	HR (nm)	ζ (mV)	PDI (%)
1	4.4	0.9	2	4547 ^e^ ± 2634	−26 ^a,b,c^ ± 2	29 ^b,c^ ± 4	2307 ^d^ ± 150	−25 ^a,b^ ± 3	39 ^a^ ± 2
2	3.6	0.3	8	132 ^b^ ± 1	−2 ^d^ ± 1	22 ^a,b^ ± 2	137 ^a^ ± 3	−10 ^b,c^ ± 7	21 ^a^ ± 1
3	4.4	0.3	8	710 ^d^ ± 12	−23 ^a,b,c^ ± 1	29 ^b,c^ ± 2	842 ^c^ ± 247	−9 ^b,c^ ± 9	30 ^a^ ± 3
4	4.4	0.9	8	265 ^a^ ± 6	−16 ^c^ ± 13	19 ^a,b^ ± 3	277 ^a,b^ ± 3	−22 ^a,b^ ± 9	23 ^a^ ± 2
5	3.6	0.9	8	135 ^b^ ± 1	−25 ^a,b,c^ ± 4	17 ^a^ ± 1	138 ^a^ ± 2	−10 ^b,c^ ± 3	22 ^a^ ± 1
6	4.4	0.3	2	9811 ^f^ ± 2298	−2.0 ^d^ ± 1	34 ^c^ ± 11	4297 ^d^ ± 749	−3 ^c^ ± 4	35 ^b^ ± 12
7	4.0	0.6	5	278 ^a^ ± 5	−31 ^a,b^ ± 3	24 ^a,b,c^ ± 2	290 ^a,b^ ± 41	−29 ^a^ ± 1	24 ^a^ ± 2
8	3.6	0.9	2	131 ^b^ ± 2	−21 ^a,b,c^ ± 2	14 ^a^ ± 3	135 ^a^ ± 4	−17 ^a,b,c^ ± 7	18 ^a^ ± 2
9	3.6	0.3	2	511 ^c^ ± 17	−18 ^b,c^ ± 2	24 ^a,b,c^ ± 10	479 ^b^ ± 17	−16 ^a,b,c^ ± 9	28 ^a^ ± 1

^a,b,c,d,e,f^ Different letters in the same column indicate significant differences between the measurements (*p* ≤ 0.05). Results presented as (mean ± standard deviation) with three replicates (n = 3). * DCA: dialyzed ι-carrageenan, HR: hydrodynamic radius, PA/PC: The volume-to-volume ratio of polyanion (PA) and polycation (PC) solutions.

**Table 5:** DLS measurements performed with suspensions (coacervates) obtained with higher polycation concentration concerning ι-carrageenan.

				25 °C			37 °C		
Assays	pH	DCA (mg/mL)	PA/PC *	HR (nm)	ζ (mV)	PDI (%)	HR (nm)	ζ (mV)	PDI (%)
10	4.4	0.9	2	509 ^c^ ± 1	+39 ^b^ ± 1	27 ^a^ ± 1	497 ^a,b,c^ ± 25	+46 ^c^ ± 1	26 ^a^ ± 1
11	3.6	0.3	8	328 ^b^ ± 22	+17 ^d^ ± 3	23 ^a^ ± 1	333 ^b,d,e^ ± 29	+3 ^a^ ± 3	28 ^a^ ± 1
12	4.4	0.3	8	396 ^a,b,^ ± 20	+48 ^b^ ± 7	21 ^a^ ± 1	359 ^a,b,d,e^ ± 23	+30 ^b,c^ ± 9	29 ^a^ ± 1
13	4.4	0.9	8	521 ^c^ ± 32	+47 ^b^ ± 7	27 ^a^ ± 2	523 ^a,c^ ± 36	+31 ^b,c^ ± 17	29 ^a^ ± 3
14	3.6	0.9	8	578 ^c,d^ ± 22	+3 ^a^ ± 0,6	30 ^a^ ± 3	577 ^c^ ± 18	+1.0 ^a^ ± 0,5	26 ^a^ ± 4
15	4.0	0.6	5	394 ^a,b^ ± 26	+16 ^c.d^ ± 2	26 ^a^ ± 2	330 ^d,e^ ± 8	+22 ^a,b^ ± 8	24 ^a^ ± 1
16	4.4	0.3	2	437 ^a^ ± 6	0 ^a^	25 ^a^ ± 3	500 ^a,c^ ± 80	+2 ^a^ ± 1	24 ^a^ ± 3
17	3.6	0.9	2	389 ^a,b^ ± 22	+12 ^a,c,d^ ± 5	26 ^a^ ± 2	382 ª^,b,d,e^ ± 8	+14 ^a,b^ ± 9	26 ^a^ ± 2
18	3.6	0.3	2	252 ^e^ ± 1	+45 ^b^ ± 8	24 ^a^ ± 1	246 ^d^ ± 2	+35 ^b,c^ ± 8	27 ^a^ ± 1

^a,b,c,d,e^ Different letters in the same column indicate significant differences between the measurements (*p* ≤ 0.05). Results presented as (mean ± standard deviation) with three replicates (n = 3). * DCH: dialyzed chitosan, HR: hydrodynamic radius, PC/PA: The volume-to-volume ratio of polycation (PC) and polyanion (PA) solutions.

**Table 6:** Particle average sizes (nm) measured from DLS and SEM.

Assays	Wet Size (HR nm) *	Dry Size (nm) **
2	132 ± 1	215 ± 61
4	265 ± 6	149 ± 53
5	135 ± 1	44 ± 17
7	278 ± 5	45 ± 11
9	511 ± 17	95 ± 26
11	328 ± 22	6.5 × 10^3^ ± 1.5 × 10^3^
12	396 ± 20	620 ± 172
15	394 ± 26	13.5 × 10^3^ ± 2.7 × 10^3^
18	252 ± 1	366 ± 93

* HR: Average hydrodynamic radius at 25 °C. ** Average dry size measured from SEM images in Figure 5.

## Data Availability

The data presented in this study are available on request from the corresponding author due to privacy or ethical restrictions.

## Supplementary Materials

The following supporting information can be downloaded at https://www.mdpi.com/article/10.3390/nano15030161/s1, Figure S1: Hydrodynamic radius measurements as a function of the pH. Figure S2: SEM images of the particles and their respective size distribution curves. The images corresponding to experiments 2, 4, 5, 7, and 9 depict coacervates prepared with an excess of ι-carrageenan (Table 1 and Table 4), while the images corresponding to experiments 11, 12, 15, and 18 depict particles obtained with an excess of chitosan (Table 2 and Table 5). Figure S3: SEM image of the particles and their respective size distribution curve. The images corresponding to experiment 7 depict coacervates prepared with an excess of ι-carrageenan (Table 1 and Table 4).
