# Supplementary material for: Iota-Carrageenan/Chitosan Nanoparticles via Coacervation: Achieving Stability for Tiny Particles
Source: Nanomaterials (Basel). 2025 Jan 22;15(3):161. doi: 10.3390/nano15030161 (PMC11819667; doi:10.3390/nano15030161)
Supplement: Supplementary file 1 [file nanomaterials-15-00161-s001.zip › nanomaterials-3390529-supplementary.pdf]

Supplementary Information

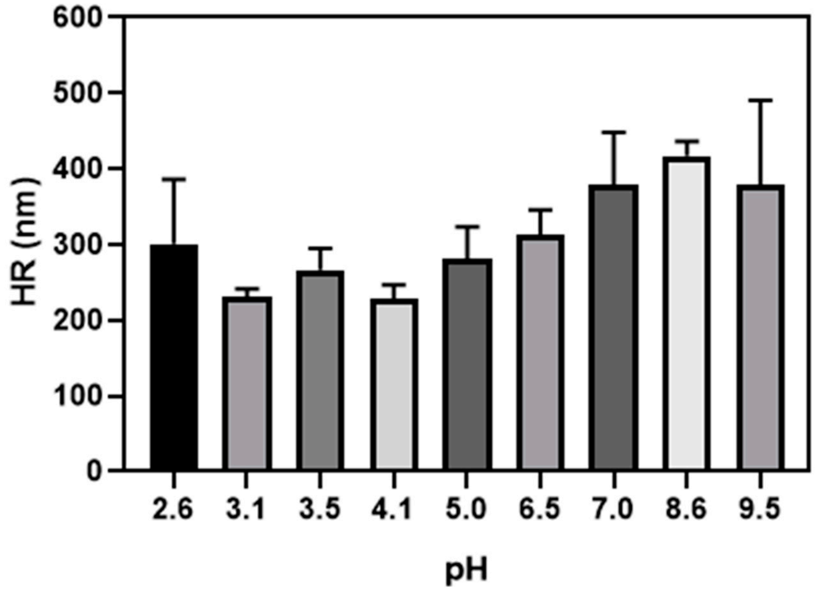

Figure S1. Hydrodynamic radius measurements as a function of pH.

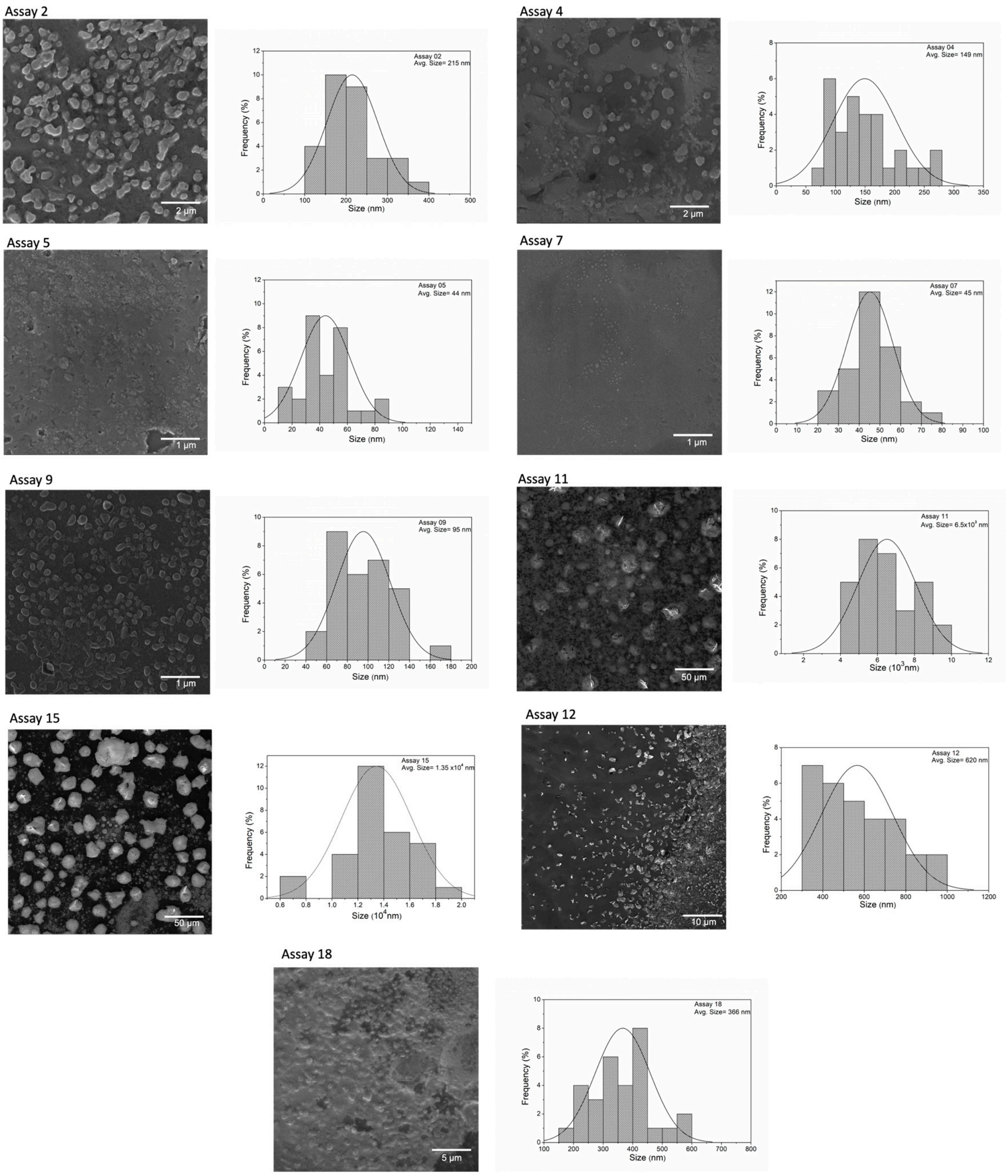

Figure S2. SEM images of the particles and their respective size distribution curves. The images corresponding to experiments 2, 4, 5, 7, and 9 depict coacervates prepared with an excess of ι-carrageenan (Tables 1 and 5), while the images corresponding to experiments 11, 12, 15, and 18 depict coacervates obtained with an excess of chitosan (Tables 2 and 6).

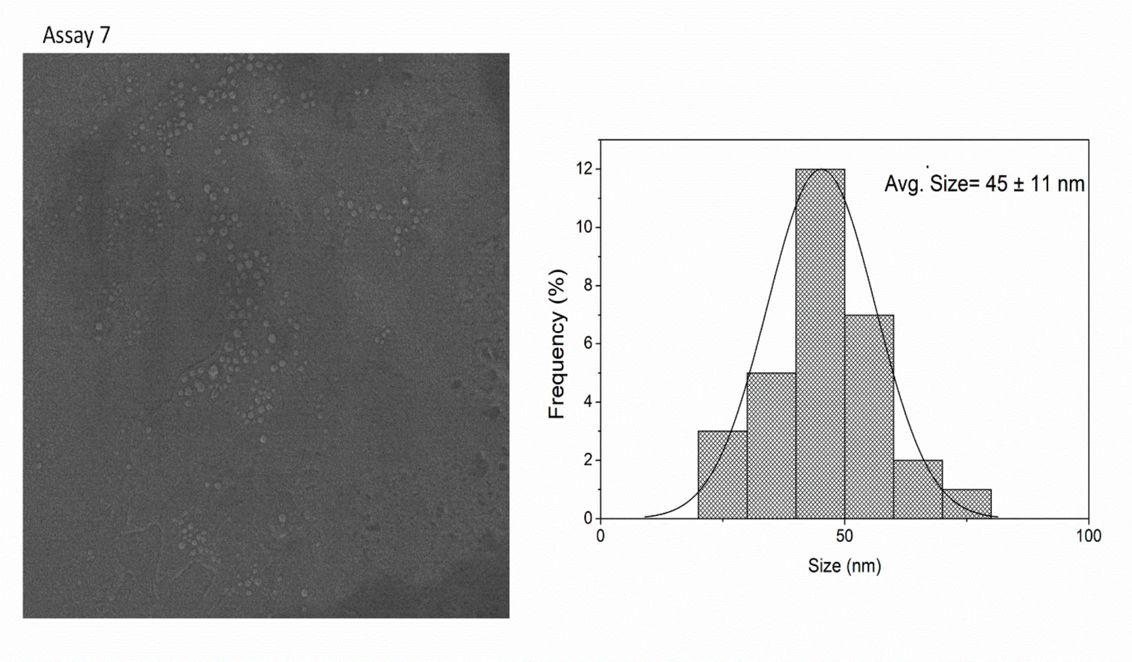

Figure 3. SEM image of the particles and their respective size distribution curve. The images corresponding to experiment 7 depict coacervates prepared with an excess of ι-carrageenan (Tables 1 and 5).
